# Salivary Exosomal MicroRNA-486-5p and MicroRNA-10b-5p in Oral and Oropharyngeal Squamous Cell Carcinoma

**DOI:** 10.3390/medicina58101478

**Published:** 2022-10-18

**Authors:** Cosmin Ioan Faur, Rareș Călin Roman, Ancuța Jurj, Lajos Raduly, Oana Almășan, Horațiu Rotaru, Magdalena Chirilă, Mădălina Anca Moldovan, Mihaela Hedeșiu, Cristian Dinu

**Affiliations:** 1 Department of Maxillofacial Surgery and Radiology, Oral Radiology, “Iuliu Hațieganu” University of Medicine and Pharmacy, 32 Clinicilor Street, 400006 Cluj-Napoca, Romania; 2 Department of Maxillofacial Surgery and Radiology, Oral and Cranio-Maxillofacial Surgery, “Iuliu Hațieganu” University of Medicine and Pharmacy, 33 Moților Street, 400001 Cluj-Napoca, Romania; 3 Research Center for Functional Genomics, Biomedicine and Translational Medicine, “Iuliu Hațieganu” University of Medicine and Pharmacy, 23 Gheorghe Marinescu Street, 400337 Cluj-Napoca, Romania; 4 Department of Prosthetic Dentistry and Dental Materials, “Iuliu Hațieganu” University of Medicine and Pharmacy, 32 Clinicilor Street, 400006 Cluj-Napoca, Romania; 5 8th Department-Surgical Secialties, O.R.L., “Iuliu Hațieganu” University of Medicine and Pharmacy, 4-6 Clinicilor Street, 400006 Cluj-Napoca, Romania; 6 Department of Maxillofacial Surgery and Radiology, Maxillofacial Surgery and Implantology, “Iuliu Hațieganu” University of Medicine and Pharmacy, 37 Iuliu Hossu Street, 400429 Cluj-Napoca, Romania

**Keywords:** head and neck neoplasms, oral malignancy, oropharyngeal cancer, salivary exosomes, microRNA, liquid biopsy

## Abstract

*Background and Objectives*: The research aimed at evaluating the capacity of salivary exosomal miR-10b-5p and miR-486-5p for oral and oropharyngeal cancer detection. *Materials and Methods*: The saliva samples were harvested from histopathological diagnosed oral and oropharyngeal squamous cell carcinoma patients and healthy volunteer subjects. The exosomes were isolated by differential ultracentrifugation and quantified by Nano Track Analysis. The microRNAs were extracted and quantified from salivary exosomes by quantitative Real-Time Polymerase Chain Reaction. *Results*: This research comprised fifty participants. When compared to healthy controls, salivary exosomal miR-486-5p was elevated and miR-10b-5p was reduced in oral and oropharyngeal squamous cell carcinoma. Moreover, miR-486-5p had a high expression level in stage II of cancer in comparison to the other cancer stages. The cancer samples presented an increased exosome dimension compared to the control samples. *Conclusions*: Salivary exosomal miR-10b-5p and miR-486-5p have an altered expression in oral and oropharyngeal cancer.

## 1. Introduction

Oral and oropharyngeal cancer (OOC) has a global incidence of 475,000 new cases in 2020 from a total of 19.1 million cancers, which are responsible for 225,000 deaths [1]. The highest common histological form of head and neck cancer (HNC) is squamous cell carcinoma (SCC), being detected in 90% of the cases [2]. Regrettably, more than half of all cancers are discovered at advanced phases (III and IV), and there is a high rate of tumor recurrence in the first two years after treatment [3,4]. Therefore, OOC is a high burden for the health care system due to its significant mortality and morbidity and lack of effective prevention or screening methods since now. A rapid and non-invasive biomarker-based method to detect OOC from the early stages of the disease is then mandatory.

Exosomes are small nanovesicles (10–150 nm) originating from cellular multivesicular bodies, different from extracellular vesicles and apoptotic bodies. Exosomes are composed of an external double layer of lipids that surrounds a cargo of proteins and nucleic acids and express surface proteins (e.g., tetraspanins, TSG101, and Alix proteins) [5,6]. The role of the exosomes is to drive information from one cell to another, ensuring cell-to-cell communication and control of the surrounding environment. Exosomes are produced by healthy and tumoral cells and they are further driven in the pericellular environment and body liquids, including blood and saliva [7]. Recently, the study of salivary exosomes and exosomal miRs gained a special interest in HNC detection and a heterogeneous population of exosomes with increased dimension and exosomal aggregates was found in cancer group samples [8].

MicroRNAs (miRs) are noncoding RNAs of small size (about twenty-two nucleotides long) present in the exosomes’ cargo, being protected from ribonuclease enzymatic degradation by the exosomes’ lipid layers [9]. The miRs attached to the 3′ untranslational region of mRNA induces degradation of mRNA or blockage of the mRNA translation into proteins, being effective controllers of gene expression [10,11]. Approximatively 60% of coding protein genes are regulated by miRs and aberrant expression of miRNAs is linked to biological pathways of human illness [12]. miR-486-5p is a muscle-enriched miRNA that is prevalent in human plasma and enriched in small extracellular vesicles. Dysregulation of miRs expression, such as increased miR-486-5p, miR-10b-5p, miR-512-5p expression, and decreased miR-200a expression were detected in HNC group samples [13,14]. MiR-486-5p was elevated at early stages in head and neck SCC patients [13]. Other miRs dysregulation as miR-4484 and miR-200 were associated with lymph node metastasis and early stage HNC detection [15,16]. Even though the exosomal miR-486-5p and miR-10-5p proved their capacity for oral and oropharyngeal cancer detection in vitro studies, we aimed to investigate their ability to detect primary cancer in a selected population and in addition, to explore the correlation between the two miRs and various tumor features, such as the tumor stage, grading, and keratinization status the correlation between the two miRs and different tumor features, such as the tumor stage, grading and keratinization status.

Saliva is an easy-to-harvest, non-invasive, and less sensitive sample compared to blood, [17,18] and most of the malignant-associated exosomes could be found in the saliva of patients with HNC [19,20]. miR-486 and miR-10b were previously identified in early-stage oral cancers and they could be valuable indicators for tumor identification and cancer spreading [13,21]. However, the relationship between the miRs and tumor stage, grading and the histopathological type has been not reported yet.

Even if the exosomal miR-486-5p and miR-10-5p their proved capacity in oral and oropharyngeal cancer detection in in vitro research and a reduced number of patients, we aimed to investigate the reliability of primary cancer detection in a larger cohort of a selected population and moreover, to explore the correlation between the two miRs and different tumor features, such as the tumor stage, grading and keratinization status.

## 2. Materials and Methods

### 2.1. Study Cohorts

Individuals assigned to the Oral and Maxillofacial Surgery and Otorhinolaryngology Departments of the Emergency County Hospital Cluj-Napoca from July 2020 to July 2021, with a histopathological diagnosis of SCC of the oral cavity or oropharynx were enrolled for this study in the cancer group. Only the patients that were negative for Human Papilloma Virus (HPV) associated infection, that further required surgical treatment, were included in this study. Volunteer subjects who were addressed to the Department of Oral and Maxillofacial Surgery for teeth extractions were comprised the control group. Medical history of cancer, previous surgical or oncological treatment as well as the presence of acute infections, were criteria of exclusion from the study. All participants completed the informed written consent form authorized by the ethics committee (nr. 461/8.11.2019). The cancer lesions were staged according to the 8th AJCC classification [22]. Demographic information was extracted from the patient ‘s healthcare documentation and the anamnesis, and the histopathological examination were provided by the Pathology Department of the Hospital.

### 2.2. Saliva Harvesting

The samples were obtained before any treatment. Participants were advised not to consume food or liquids starting the night before and not perform oral hygiene on the morning of saliva collection. A protocol of unstimulated saliva harvesting was applied in the morning, between 7 a.m. and 10 a.m., 15 min after the oral cavity was rinsed with water [23,24,25,26]. To obtain only unstimulated saliva samples, the patients were asked to passively drool saliva into a sterile receptacle particularly designed for saliva harvesting and not to cough or aggressively spit the material. A quantity of 0.8 to 1.6 mL of saliva was collected. The samples were deposited in a refrigerator at −20 °C for a maximum of 1 week and then they were transferred for depositing at −80 °C until further processing.

### 2.3. Exosome Isolation and Characterization

The following materials were used for exosomal miRs separation and characterization in the saliva samples: PBS PH7.4 with and without Ca si Mg (Invitrogen, Waltham, MA, USA), Plasma/Serum Circulating and Exosomal RNA Purification Kit (Norgen Biotek Corp, Thorold, ON, Canada), PowerUp™ SYBR™ Green Master Mix (Applied biosystem, Thermo Fisher Scientific, Waltham, MA, USA), TaqMan Fast Advanced Master MIX (Applied biosystem, Thermo Fisher Scientific, Waltham, MA, USA), TaqMan™ MicroRNA Assay (Invitrogen, Thermo Fisher Scientific, Waltham, MA, USA), TaqMan MicroRNA Reverse Transcription Kit (Applied biosystem, Thermo Fisher Scientific, Waltham, MA, USA) and RNase inhibitor (Applied biosystem, Thermo Fisher Scientific, Waltham, MA, USA). 

The sample analysis started with exosome isolation by a differential ultracentrifugation protocol [13,15,23]. Briefly, saliva samples were slowly thawed at room temperature. The samples were firstly centrifuged at 4 °C and 12.000× *g* for 20 min to remove cells and cellular detritus. The supernatant was collected, diluted 1:1 with PBS 1×, and then filtered through a 0.22μm filter. Further, the filtered supernatant was ultracentrifuged at 120.000× *g*, 4 °C for 70 min. The pellet of exosomes was rinsed with 1× PBS and ultracentrifuged at 120.000× *g*, 4 °C, lasting one hour and ten minutes. In the end, exosomes were located at the bottom of the centrifugation tube. They were extracted from the last 300 μL of liquid located at the base of the tube. Exosomes were stored at −80 °C until the next step of the analysis.

The purified exosomes were diluted 10× with PBS 1× and further analyzed for quantification and characterization using the NanoSight NS300 instrument (NanoSight Limited, London, UK). The particle-size distribution of each particle was determined according to their Brownian motion. A 60-s video with a frame rate of 30 frames/s was captured, and the NanoSight Nano Track Analysis (NTA) 3.2 program was used to analyze particle movement.RNA extraction

Saliva exosome specimens were collected and purified employing a Plasma/Serum Circulating and Exosomal RNA Purification Kit according to the manufacturer’s instructions. (Thermo Fisher Scientific, Waltham, MA, USA) and the extracted RNA was diluted accordingly to reach 25 ng in the cDNA reaction mix.

### 2.4. cDNA Synthesis and qRT-PCR

The cDNA synthesis was performed according to the manufacturer protocol, using TaqMan MicroRNA Reverse Transcription Kit (https://www.thermofisher.com/order/catalog/product/4366596 (accessed on 16 August 2022)).

Quantitative Real-Time Polymerase Chain Reaction (qRT-PCR) was conducted using a total volume of 10 μL and 5 μL of cDNA (adjusted 1:3 with nuclease-free water), 4.5 μL of TaqMan Fast Advanced Master MIX, and 0.73 μL primer for each miRNA in ViiA7 PCR machine (Applied biosystem, Thermo Fisher Scientific, USA, Waltham, Massachusetts). The primers of qRT-PCR were listed in Table 1. The reactions were set up as follows: initial denaturation step at 50 °C for 120 s and 95 °C for 2 s, followed by 40 cycles of 95 °C for 1 s and 60 °C for 20 s. The expression level of each miRNA was determined using the threshold cycle method (CT). The relative expression level was determined through 2^−ΔΔCT^. miR-16 was employed as an internal standard for miRNA expression.

Statistic assessment The GraphPad Prism 6 software was employed for statistical evaluation. Categorical variables were represented as an absolute value (percent). Demographical and clinical features of the population were assessed by student *t*-test, and they were represented by mean ± standard deviation. The miR-10b-5p and miR-486-5p patterns for head and neck SCC patients were searched in the Cancer Genome Atlas dataset (TCGA-UALCAN platform) [27]. The logarithm of relative fold changes (LR fold changes) for miRS expression was calculated. Fisher’s exact test was used to examine the tables of contingencies. The distribution’s normality was evaluated using the Shapiro-Wilktest and histogram interpretation. All continuous variables of exosomes and miRs were non-normally distributed; thus, we assessed the differences between the two groups using the Mann-Whitney-Wilcoxon rank sum test. Differences between more than two non-normally distributed groups were assessed using Kruskal-Wallis’s test. Non-normally distributed variables were represented as median (quartile 1, quartile 3). A p result of less than 0.05 was regarded as statistically relevant. The capacity of miRs to predict cancer presence in saliva samples based on exosome analysis was evaluated by Area Under the Receiver Operating Characteristics and presented as Area Under the Curve (AUC).

## 3. Results

This research recruited fifty participants (25 patients in the malignancy sample and 25 controls). The mean age was 59 ± 9 years in the tumor sample and 54 ± 14 years in the controls, with no statistically significant difference between them. The cancer group included 22 males and 3 females, and the control group enrolled 12 males and 13 females. In terms of gender, there was a statistical distinction among the groups (*p* < 0.01). The histological grading showed 6 patients with well-differentiated (G1) SCC, 14 patients with intermediate-differentiated (G2) SCC, and 5 patients with poorly differentiated (G3) SCC. A number of 20 keratinized SCC and 5 non-keratinized SCC were found. Most of the primary tumors were diagnosed in stage IV (17 patients), followed by stage III (4 patients) and stage II (4 patients). There were 21 patients with oral cavity cancers and 4 patients with oropharyngeal cancer.

### Exosome Isolation and Characterization

**Figure 1:**
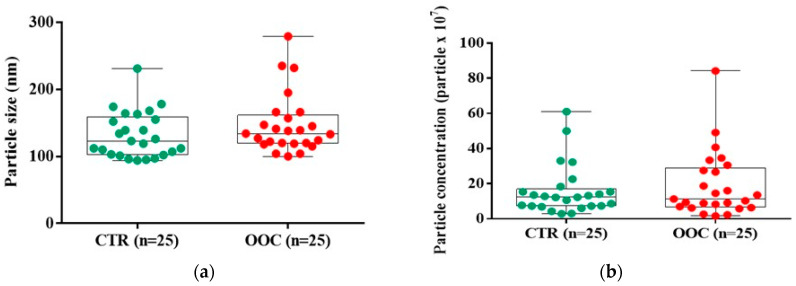
Characterization of salivary-derived exosomes isolated from healthy and cancer groups through ultracentrifugation. (**a**) Size distribution profiles of exosomes were determined based on their Brownian motion using NanoSight. (**b**) The concentration of salivary-derived exosomes based on the particle concentration (particles × 107). Data were analyzed using Mann-Whitney-Wilcoxon rank sum test. The miRs identification and expression level.

Exosomes were discovered in every sample evaluated by the NanoSight device, however, there was no statistically significant demarcation between the OOC sample of participants and healthy participants However, the overall data demonstrated that the tumor sample’s exosome population was more heterogeneous and larger (median (Q1, Q3): 134 (120, 157) nm) compared with the control group (123 (103, 155) nm) (Figure 1A). A lower concentration of exosomes was identified in the cancer group (11.3 (7.0, 27.5) particles/μL × 107) compared to the control group (12.3 (7.3, 15.4) particles/μL × 107) (Figure 1B).

**Figure 2:**
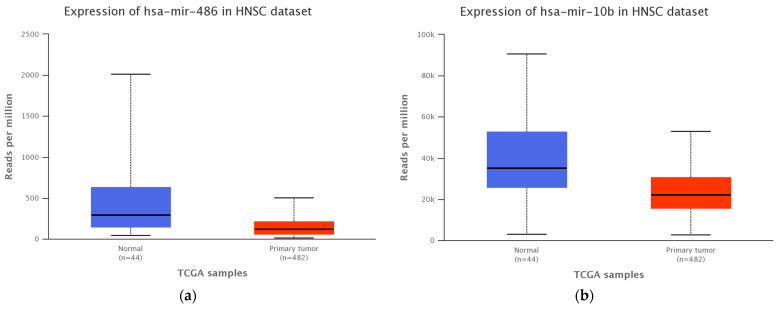
Expression level of (**a**) miR-10b-5p (*p* = 0.0001) and (**b**) miR-486-5p (*p* = 0.0002) in TCGA head and neck SCC patients cohort and healthy control group.

The miRs pattern for head and neck SCC patients from the Cancer Genome Atlas dataset (TCGA-UALCAN platform) revealed an altered expression level for miR-486-5p and miR-10b-5p analyzed miRs switch, that was further validated in the patient cohort included in our study (Figure 2).

The miR-486-5p and miR-10b-5p signal was detected in 24 exosomes samples of OOC and 25 exosomes samples of control healthy subjects. One sample in the OOC group cannot be evaluated to quantify the miR expression.

**Figure 3:**
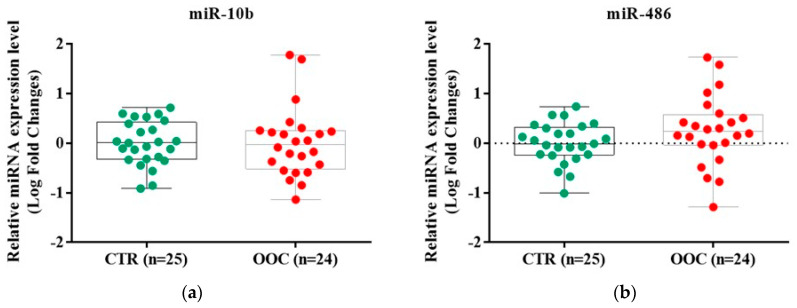
The evaluation of microRNAs expression profile in cancer and healthy samples using RT-qPCR method; (**a**) Difference between the expression level of miR-10b-5p in cancer and control groups; (**b**) Difference between the expression level of miR-486-5p in cancer and control groups. The data were normalized to U6 and RNU48 using the ΔΔCT method. Data were analyzed using Mann-Whitney-Wilcoxon rank sum test and represented as Log of Fold Change. The miR-10b-5p expression level presented no difference between oral cavity cancer (1.38 (0.35, 1.75 LR fold changes) and oropharyngeal cancer (1.38 (0.35, 1.75) LR fold changes). The miR-486-5p expression levels were elevated in in oropharyngeal cancer (2.11 (1.90, 2.33) LR fold changes) compared to oral cavity cancer (1.44 (0.80, 4.48) LR fold changes). The results were not statistically significant.

The exosomal miRs expression which was evaluated by qRT-PCR indicated a different relative expression level between cancer patients and the healthy subjects’ group (Figure 3). The miR-10b-5p was downregulated in 14/24 samples of the cancer group (0.96 (0.35, 1.75) LR fold changes) compared with healthy controls (1.02 (0.48, 2.48) LR fold changes), with no statistical significance between the groups. The miR-486-5p was upregulated in 15/24 samples of the cancer group (1.74 (0.95, 3.43) LR fold changes) compared with the control group (0.98 (0.59, 2.01) LR fold changes), without any statistical significance.

Table 2 summarizes the miRs expression according to the tumor stage, grading, and keratinizing status. Stage II had a higher upregulation of miR-486-5p expression than stage III or Stage IV. On the other hand, the miR-10b-5p was decreased in stage IV of the disease and increased in stages II and III. No statistical difference was found between miRs expression of the tumor stages.

**Figure 4:**
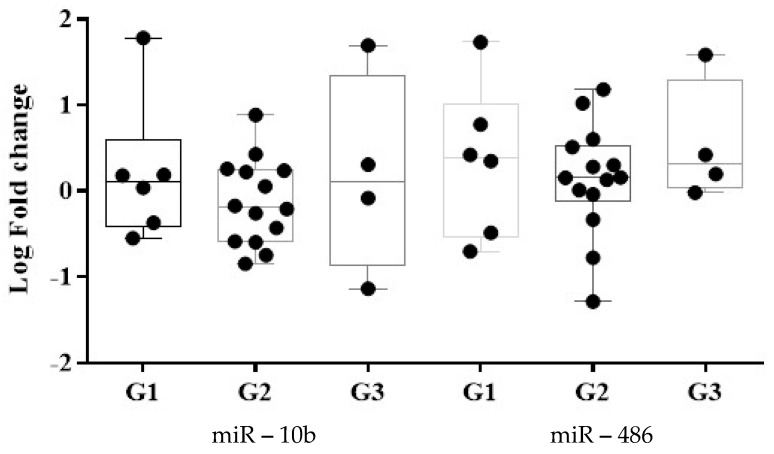
Tumor grading classification using the expression level of miR-486-5p and miR-10b-5p. Data were analyzed using Mann-Whitney-Wilcoxon rank sum test and represented as Log of Fold Change. (G1 = well-differentiated; G2 = intermediate-differentiated; G3 = poorly differentiated) ROC curves were built to assess the discriminatory capacity of the two dysregulated miRNAs as possible biomarkers for OOC detection The ROC curves express the sensitivity and specificity for each miRNA, the AUC indicating the discrimination power of the biomarker (Table 3). The miR-486-5p and miR-10b-5p exhibited relative AUCs (standard error; 95% confidence interval; *p* values) of 0.72 (0.07; 0.57, 0.88; 0.01) and 0.59 (0.08; 0.43, 0.76; 0.26) for malignancy identification. The miR-486-5p had a higher AUC in oropharynx cancer detection compared with oral cavity cancer. Furthermore, compared to stages III and IV, the miR-486-5p exhibited a greater cancer detection AUC in stage II and has the highest AUC for G3 and non-keratinized SCC identification. The miR-10b-5p had the greatest AUC OOC detection in stage IV of the disease and oral cavity cancer sites. The G2 and non-keratinized SCC presented a higher AUC compared with the other histopathological type of the OOC.

A different expression of miRs related to the OOC grade was identified. miR-486-5p expression was observed to be greater in G1 SCC relative to G3 SCC (Figure 4). The miR-10b-5p presented a downregulation in G2 and upregulation in G1 and G3. The miRs were expressed differently in non-keratinizing SCC tumors: miR-10b-5p was downregulated and miR-486-5p was upregulated.

## 4. Discussion

The findings revealed alteration of exosomal miR-486-5p and miR-10b-5p expression in OOC patients’ salivary specimens. Even in stage II of oral and oropharyngeal carcinoma, salivary exosomal miR-486-5p was highly expressed. Exosomes may also act as independent OOC biomarkers [21,28] In saliva samples from cancer patients, we discovered an increased dimension and heterogeneous population of exosomes. These alterations could be explained by the intense metabolism of the malignant tumors that releases a high amount of exosomes into the environment to promote tumor spreading [11]. These results are relevant due to the lack of studies on non-invasive molecular biomarkers of oral and oropharyngeal SCC and the small samples included since now [21].

Owing to the varied expression of exosomal miRs and saliva alterations in the elderly compared to young individuals [25,29], age-matched healthy subjects have been chosen for the control group. The preponderance of the tumor sufferers in the present research (84%) had progressed disease (stage III or IV), the study is performed during COVID-19 pandemic lockdowns that made access to medical care more difficult [30,31].

Our findings were consistent with other studies that found an upregulation of the miRs and an AUC value for miR-412-3p in OOC detection that ranged between 0.73 and 0.87. As well to the salivary exosomal miR-24-3p, miR-512-3p and miR-412-3p overexpression in OOC, the tongue tissue non-exosomal miR-486-5p proved upregulation in OOC [8,14,32]. The overall results demonstrate the good reliability of salivary miRs for OOC detection.

For OOC detection, Langevin et al. reported high specificity (100% for miR-10b-5p and 89% for mir-486-5p) and low the sensitivity (18% for miR-10b-5p and 45% for mir-486-5p) with 2 distinct cut-off thresholds for miR-10b-5p and mir-486-5p, respectively (cut-off point of >1.0 copies/L and >100 copies/L) [13]. In our study, we identified an overexpression of miR-486-5p relative to the control sample, with a 2-folds higher expression in oropharyngeal cancer and with a 1,5-fold higher expression in oral cavity cancer. Along with miR-486-5p overexpression, oropharyngeal cancer also had a higher AUC value for cancer detection (AUC value of 0.89) than oral cavity cancer (AUC value of 0.67). As a result, salivary exosomal miR-486-5p may be a possible site-specific biomarker for SCC of the oropharynx.

Salivary miR-486-5p level was considerably overexpressed in OOC stage II compared to advanced stages (III or IV) of cancer and a high AUC value (0.76) for stage II carcinoma identification was found. Similar to earlier findings published by Langevin et al., these results reveal that miR-486-5p may be a viable diagnostic potential for initial-stage OOC identification [13].

The contrast between exosomal miR-10b-5p’s enhanced expression in stages II and III and decreased expression in stage IV is an intriguing finding. An explanation could be the dysregulation of miRs associated with an increased genetic activity that promotes malignant cell invasion and dissemination [33,34]. Although the various pattern of miRs expression in different stages of cancer have been described, additional fundamental studies are required to fully understand the way they can predict tumor spreading and the evolution of the disease [35].

Non-keratinizing and G1 tumors have a better 5-years survival rate compared to keratinizing and G3 SCC [36,37,38]. In our study, different alterations of the miRs expression were associated with distinct histopathological grades and keratinization variations. In contrast to the highest AUC value of miR-485-5p upregulation in G3 SCC, the maximum AUC value for miR-10b-5p downregulation was in G2 SCC. Although both miRs had the greatest AUC value for non-keratinizing SCC tumors, miR-486-5p was upregulated and miR-10b was downregulated. However, a relationship between different exosomal miRs expression and SCC grades have been identified in previous studies [39]. The exosomal miR-134 overexpression was associated with G1 SCC, whereas the exosomal miR-200a overexpression was related to G3 cancer, in saliva samples (G3) [40]. This is, as far as we are aware, the first study to demonstrate a correlation between salivary exosomal miRs and the keratinization state of OOC.

A potential shortcoming of our research was the small amount of individuals with OOC, even if it was higher than in other studies [21]. To minimize the variability of the results due to different secretion of biochemical salivary compounds during the day, saliva collection was performed in the morning [40,41]. The same saliva harvesting technique previously reported for the examination of the genetic material was used for our samples. We also used differential ultracentrifugation for exosome isolation due to the heterogeneity of the results brought on by the affinity of filter base techniques [42]. Additionally, due to financial constraints, only NanoSight was employed to establish the existence of exosomes in saliva samples with a range of size detection between 10 and 150 nm [43,44].

In contrast to Langevin et al., who employed droplet digital PCR, we quantified the miRs activity in salivary exosomes using qRT-PCR. In addition, we queried the UALCAN platform to investigate the miR-10b-5p and miR-486-5p pattern for patients with head and neck SCC and UALCAN screening indicated altered expressions of miR-10b-5p and miR-486-5p in head and neck SCC [27,45].

Exosomes are sensitive nanovesicles, difficult to isolate from any samples, and even more difficult to evaluate in saliva samples. Moreover, the exosomal miRs copies are heterogeneous presents in the exosomes population and the quantification process requires a high number of exosomes that can offer a high number of miRs [46,47]. The difficulty to assess the exosomal miRs and a limited number of patients could explain the lack of statistical significance of our results. However, the exosomal miRs improve the accuracy of SCC detection owing to the particular origin of exosomes released by the cancer cells, by excluding the cell-free miRs from desquamated and dead cells [48,49]. Also, tobacco smoking can induce dysregulation of miRs expression related to increased cell scattering and invasiveness of oral keratinocytes [50]. Future research is still required to examine how smoking affects the dysregulation of miR-10b-5p and miR-486-5p in HNC.

The Human Papilloma Virus (HPV) may induce changes in oral and oropharyngeal SCC miRs biomarkers [51]. A limitation of our study was the lack of investigation of the correlation between HPV-associated infection and the changes in exosomal miRs due to the lack of included patients. However, further research is needed.

## 5. Conclusions

Both miR-486-5p and miR-10b-5p dysregulations in salivary exosomes have been associated to OOC, with miR-486-5p being overexpressed in stage II of SCC. Exosomal miRs expression levels in salivary samples are influenced by the keratinization and differentiation grade of the tumor. Our study revealed that saliva could be a suitable biological sample for potential biomarkers of OOC, such as exosomal miRs. However, more studies are required to analyze the correlation between salivary exosomal miRs and oral and oropharyngeal SCC.

## Figures and Tables

**Table 1:** miRNA primers used in the study.

Assay Name	Assay ID	miRNA Sequence
hsa-miR-16-5p	000391	UAGCAGCACGUAAAUAUUGGCG
hsa-miR-10b-5p	002218	UACCCUGUAGAACCGAAUUUGUG
hsa-miR-486-5p	001278	UCCUGUACUGAGCUGCCCCGAG

**Table 2:** miR-10b-5p and miR-486-5p expression (Logarithm of relative fold changes).

	miR-10b-5p					miR-486-5p				
	Median	Q1	Q3	IQR	*p*	Median	Q1	Q3	IQR	*p*
Keratinized SCC	1.09	0.49	1.92	1.43	0.33	2.01	1.58	3.24	1.66	0.93
Non-keratinized SCC	0.37	0.26	1.66	1.40		1.44	0.94	4.29	3.35	
Stage II	5.19	2.05	20.85	18.80	0.13	12.85	7.85	24.81	16.96	0.33
Stage III	1.47	1.02	1.87	0.85		1.67	1.42	2.09	0.67	
Stage IV	0.59	0.28	1.53	1.25		1.51	0.80	2.80	2.00	
Well-differentiated G1 SCC	1.31	0.59	1.54	0.95	0.74	2.44	0.80	5.11	4.31	0.64
Intermediate-differentiated G2 SCC	0.65	0.29	1.71	1.42		1.44	0.94	2.93	1.99	
Poorly differentiated G3 SCC	1.43	0.64	13.86	13.22		2.11	1.42	11.59	10.17	

Abbreviations: Q1: quartile 1; Q3: quartile 3; IQR: interquartile range.

**Table 3:** Area under the curve of oral and oropharyngeal cancer detection using miR-486-5p and miR-10b-50.

	miR-486-5p				miR-10b-5p			
	AUC	Standard Error	95% Confidence Interval	*p*-Value	AUC	Standard Error	95% Confidence Interval	*p*-Value
Oral cavity	0.67	0.08	0.50, 0.84	0.05	0.58	0.09	0.41, 0.76	0.33
Oropharynx	0.89	0.06	0.76, 1.02	0.01	0.50	0.16	0.18, 0.82	0.97
Stage II	1	0	1.00, 1.00	<0.01	0.61	0.23	0.15, 1.08	0.51
Stage III	0.75	0.09	0.56, 0.93	0.11	0.61	0.11	0.38, 0.84	0.47
Stage IV	0.58	0.09	0.39, 0.77	0.36	0.68	0.08	0.51, 0.85	0.05
Well-differentiated G1 SCC	0.71	0.17	0.36, 1.07	0.16	0.55	0.13	0.29, 0.82	0.68
Intermediate-differentiated G2 SCC	0.66	0.09	0.47, 0.85	0.10	0.61	0.09	0.42, 0.80	0.25
Poorly differentiated G3 SCC	0.79	0.11	0.57, 1.01	0.06	0.55	0.19	0.17, 0.92	0.74
Keratinized SCC	0.71	0.08	0.54, 0.87	0.02	0.54	0.09	0.35, 0.72	0.66
Non-Keratinized SCC	0.85	0.07	0.70, 1.00	0.02	0.67	0.14	0.38, 0.96	0.22

Abbreviation: AUC: area under the curve; SCC: squamous cell carcinoma.

## Data Availability

The data described in this research are accessible on fair inquiry through the primary contributor.
